# Sperm Oxidative Stress during In Vitro Manipulation and Its Effects on Sperm Function and Embryo Development

**DOI:** 10.3390/antiox10071025

**Published:** 2021-06-25

**Authors:** Roberto Gualtieri, Guruprasad Kalthur, Vincenza Barbato, Salvatore Longobardi, Francesca Di Rella, Satish Kumar Adiga, Riccardo Talevi

**Affiliations:** 1 Department of Biology, University of Naples “Federico II”, Complesso Universitario di Monte S. Angelo, Via Cinthia, 80126 Naples, Italy; barbato_vincenza@libero.it (V.B.); riccardo.talevi@unina.it (R.T.); 2 Department of Clinical Embryology, Kasturba Medical College, Manipal Academy of Higher Education, Manipal 576 104, India; guru.kalthur@manipal.edu (G.K.); satish.adiga@manipal.edu (S.K.A.); 3 Global Clinical Development Fertility and Endocrinology, Merck Healthcare KgaA, Frankfurter Strasse 250, 64293 Darmstadt, Germany; salvatore.longobardi@merckgroup.com; 4 UOC Oncologia Clinica Sperimentale di Senologia, Istituto Nazionale per lo Studio e la Cura dei Tumori, Fondazione G. Pascale, 80131 Naples, Italy; f.dirella@istitutotumori.na.it

**Keywords:** spermatozoa, male infertility, oxidative stress, assisted reproductive technologies, embryo development

## Abstract

Reactive oxygen species (ROS) generated at low levels during mitochondrial respiration have key roles in several signaling pathways. Oxidative stress (OS) arises when the generation of ROS exceeds the cell’s antioxidant scavenging ability and leads to cell damage. Physiological ROS production in spermatozoa regulates essential functional characteristics such as motility, capacitation, acrosome reaction, hyperactivation, and sperm-oocyte fusion. OS can have detrimental effects on sperm function through lipid peroxidation, protein damage, and DNA strand breakage, which can eventually affect the fertility of an individual. Substantial evidence in the literature indicates that spermatozoa experiencing OS during in vitro manipulation procedures in human- and animal-assisted reproduction are increasingly associated with iatrogenic ROS production and eventual impairment of sperm function. Although a direct association between sperm OS and human assisted reproductive techniques (ART) outcomes after in vitro fertilization (IVF) and/or intracytoplasmic sperm injection (ICSI) is still a matter of debate, studies in animal models provide enough evidence on the adverse effects of sperm OS in vitro and defective fertilization and embryo development. This review summarized the literature on sperm OS in vitro, its effects on functional ability and embryo development, and the approaches that have been proposed to reduce iatrogenic sperm damage and altered embryonic development.

## 1. Introduction

Infertility, defined by the World Health Organization (WHO), as the inability to achieve pregnancy within 12 months of regular unprotected sexual intercourse, affects approximately 15% of couples, out of which a male factor is responsible for approximately half of the cases [1,2]. The causes of male infertility are numerous, and includes genetic causes such as Y-chromosome deletions in severe oligozoospermic and non-obstructive azoospermic men, varicocele, infections of the male reproductive tract, presence of anti-sperm antibodies, non-obstructive and obstructive azoospermia, and hypogonadism [3]. About 15% of infertile men have unexplained infertility as no defects are observed after routine semen analysis [4]. Moreover, 30–50% of male infertility cases are idiopathic as routine semen analysis has no identifiable etiology, and a female factor seems to be absent [5,6,7]. Indeed, the incidence of male infertility oscillates in a wide range (5–35%) due to multifactorial differences among the patient’s populations [8,9].

Although it is widely accepted that a semen profile based on sperm number, motility, and morphology according to the WHO is fundamental in the evaluation and treatment of the infertile couple, its predictive value on the fertilizing ability, either in vivo or in vitro, is limited. Oxidative stress (OS) and sperm DNA damage have shown a strong association with several forms of male infertility. In fact, about 30–80% of infertile men have elevated levels of reactive oxygen species (ROS) and lower antioxidant capacity in their ejaculate [10,11,12]. Hence, the term Male Oxidative Stress Infertility (MOSI) has been recently introduced to describe infertile men with abnormal semen characteristics and OS, including many patients previously classified as infertile idiopathic males [13]. Indeed, controlled production of ROS in spermatozoa has a physiological role in redox signaling and drives fundamental events in spermatozoa such as the activation of motility, transient sperm-oviduct adhesion, capacitation, hyperactivation, acrosome reaction, and sperm oocyte fusion [14,15,16,17,18]. On the other hand, when semen ROS levels exceed the antioxidant’s defense, a status of oxidative stress is generated. OS plays a crucial role in a wide range of diseases, including infertility, and is also clearly influenced by environmental and lifestyle factors. It may impair not only female and male reproductive health but also exert epigenetic effects on the offspring [19]. Spermatozoa are vulnerable to OS due to their high content of polyunsaturated fatty acids (PUFA), deficiencies in intracellular antioxidant enzymes, and limited DNA repair ability. ROS cause impairment in male fertility by damaging the sperm membrane via lipid peroxidation with consequent reduction of sperm motility, sperm-oocyte fusion [20,21], and the sperm DNA integrity, thus compromising the paternal genomic contribution to the embryo [22,23,24,25]. Sperm DNA damage, in most cases is believed to be oxidative in nature and associated with reduced fertilization rates, impaired preimplantation development, increased incidence of miscarriage, and fetal loss [11,26,27,28,29,30,31]. Although several mechanisms are known to induce DNA damage during spermatogenesis and epididymal maturation, sperm DNA can also be damaged post-ejaculation. 

ART outcome, including fertilization and clinical pregnancy rates, are influenced by multiple factors, among which sperm OS plays a significant role. Sperm oxidative DNA damage, if left unrepaired after fertilization, can compromise embryo development or transmit genetic mutations to the offspring [9]. During in vitro handling, spermatozoa experience altered microenvironments, shearing forces, and a wide spectrum of exogenous factors which are different from the specific physicochemical microenvironments they sequentially encounter during their journey within the female reproductive tract. The regulation of ROS levels within physiological concentrations during sperm handling in ART represents a crucial factor in optimizing its clinical efficiency. The laboratory interventions such as sperm handling, washing techniques, and cryopreservation could generate or increase the status of OS in spermatozoa, and this could be a concern, especially when ROS levels and/or DNA damage in the basal semen are elevated.

This manuscript aims to review the literature on sperm OS during in vitro handling in ART, its effects on sperm function and embryo development, and the remedies that have been proposed to reduce iatrogenic sperm damage and impairment of embryo development.

## 2. Sources of ROS in Spermatozoa

Various sources in spermatozoa produce ROS such as superoxide (O_2_^•−^) [22,32], hydrogen peroxide (H_2_O_2_) [22], nitric oxide [33], and peroxynitrite [34]. Mitochondria represent one of the main ROS sources in spermatozoa [18]. Production of mitochondrial ROS is due to the leakage of electrons from the electron transport chain (ETC), which are then accepted by molecular oxygen (O_2_) producing O_2_^•−^ [35,36]. ROS also results from the mitochondrial apoptotic pathway, which has been reported to be activated by the dysfunction of the phosphoinositide signaling pathway [37]. Moreover, mitochondrial membranes are rich in PUFA that represent preferential substrates for ROS, triggering lipid peroxidation and the generation of highly reactive lipid aldehydes. These covalently bind to ETC proteins, reinforcing the production of mitochondrial ROS in a self-perpetuating cycle, and compromise both the competence and the DNA integrity of the spermatozoa [38,39,40]. The fact that unesterified unsaturated fatty acids are powerful ROS inducers in human spermatozoa also indicates a role for lipoxygenase in this process [41]. 

Several lines of evidence in different species support the role of the hexose monophosphate shunt pathway in ROS generation by spermatozoa [42,43]. In human spermatozoa, ROS production has been highly correlated with the presence of glucose-6-phosphate-dehydrogenase, a key enzyme in the control of hexose monophosphate shunt activity [44]. Among NADPH oxidases involved in ROS production in phagocytes [45], a novel isoform of NADPH oxidase 5 (NOX5) localized in the membrane, activated by binding of calcium ions (Ca^2+^) and involved in the production of O_2_^•−^ and H_2_O_2_, has been detected in the acrosome, neck, and tail of human spermatozoa [46,47]. Nitric oxide synthase and other non-enzymatic mechanisms have also been proposed to participate in the production of nitric oxide [48,49].

## 3. Roles of ROS in Sperm Physiology

Ejaculated spermatozoa move along the female reproductive tract until they reach the oviductal ampulla to fertilize the oocyte. Redox signaling through the production of low levels of ROS is involved in several sperm physiological processes such as transient sperm-oviduct adhesion, capacitation, hyperactivation, acrosome reaction, and the membrane fusion with the oocyte. The two main events that orchestrate the physiological changes associated with sperm capacitation are the activation of Src family kinases with the consequent inhibition of serine/threonine (Ser/Thr) phosphatases and the activation of the cAMP pathways by bicarbonate and Ca^2+^. Downstream changes such as the increase in intracellular pH and membrane hyperpolarization have been hypothesized to increase intracellular Ca^2+^ through the modulation of specific sperm channels. Such an increase is essential for inducing sperm hyperactivation, acrosome reaction, and developing the ability to fertilize the oocyte [50]. Redox regulation of the thiol groups of cysteine residues is necessary to regulate different sperm proteins associated with capacitation [51]. In several species, spermatozoa transiently adhere to the epithelial cells lining the isthmic portion of the oviductal epithelium [52]. This adhesion selects high-quality spermatozoa and maintains their fertilizing ability until periovulatory release, which allows their migration toward the oviductal ampulla for fertilization [53]. Several pieces of evidence indicate that selected spermatozoa are not capacitated, and that capacitation allows their release from the oviductal reservoir. The quantity of sperm surface thiols has been shown to increase during capacitation [51], and the level of thiols in sperm-surface proteins modulates the ability of bovine spermatozoa to adhere to and be released from the oviduct in vitro and their capacitation status [15,17]. 

Concentrations of O_2_^•−^, H_2_O_2_, nitric oxide, and peroxynitrite are progressively increased during the entire course of capacitation [32,54]. An early event of capacitation regulated by ROS is the activation of adenylate cyclase by O_2_^•−^ and nitric oxide, which drives the increase of intracellular cyclic adenosine monophosphate (cAMP), followed by the activation of protein kinase A (PKA), which is essential for the tyrosine phosphorylation of target proteins [55,56]. Later capacitation molecular events in which specific ROS are implicated include the mitogen-activated protein kinase (MEK), extracellular-regulated kinase (ERK), phosphoinositide-3 kinase/Akt (PI3K/Akt) pathways, and tyrosine phosphorylation [57,58,59,60].

Hyperactivation, a high amplitude, increased lateral head displacement, asymmetric flagellar movement, is part of capacitation and is required for successful sperm passage through the cumulus oophorus and the zona pellucida and for fertilization. This motility change depends on ROS-mediated tyrosine phosphorylation of flagellar proteins, and the simple exposure of capacitating spermatozoa to ROS increases hyperactivation [61,62].

The increase of membrane fluidity needed for induction of acrosome reaction and sperm-oocyte fusion is influenced by ROS. In fact, exposure of spermatozoa from several species to exogenous H_2_O_2_ was shown to induce both the acrosome reaction and the sperm-oocyte fusion [63,64,65]. In particular, exposure of human spermatozoa to H_2_O_2_ induces the acrosome reaction whereas, this is inhibited by the addition of catalase [66,67]. 

## 4. Effects of Oxidative Stress on Sperm Function

Spermatozoa are vulnerable to OS due to their high content of PUFA, deficiencies in intracellular antioxidant enzymes, and limited DNA repair ability. In particular, the high content of PUFA in the sperm plasma membrane regulates membrane fluidity but also represents a preferential substrate for ROS attack. In fact, lipid peroxidation of the sperm plasma membrane PUFA has been the first oxidative damage recognized in male infertility [22,68]. The highly reactive lipid aldehydes produced by peroxidation form adducts with proteins and DNA and induce sperm mitochondrial dysfunction through binding to ETC proteins. This reinforces the production of mitochondrial ROS and compromises both the competence and the DNA integrity of the spermatozoa [38,39,40], causing mutations in the sperm genome [9]. The generation of 4-hydroxynonenal-protein adducts has been associated with loss of membrane integrity, motility, and reduced fertility [69].

## 5. Origin and Consequences of DNA Damage in Ejaculated Spermatozoa

Three main mechanisms are currently considered for the induction of DNA damage in spermatozoa: abortive apoptosis during spermatogenesis [70], defective protamination during spermiogenesis, defective epididymal microenvironment, and OS. Among these, OS is considered to be the major cause of spermatozoa [24]. In fact, high ROS levels and decreased total antioxidant capacity are closely correlated to increased sperm DNA damage in infertile men [71,72,73,74]. ROS can directly damage DNA, generating oxidized DNA adducts such as 8-hydroxy-2-deoxyguanosine (8-OHdG), 1,N6-ethenoadenosine, and 1,N6-ethenoguanosine. In particular, 8-OHdG, caused by hydroxyl radicals, represents the most frequently generated adduct and is assessed as a marker of DNA oxidative damage in several studies [25,75]. 

Defective protamination renders the spermatozoa highly susceptible to oxidative DNA damage and DNA fragmentation [75]. DNA bases in the nucleus and mitochondria can also be deaminated, nitrated, or oxidated by nitric oxide [76]. 8OHdG is highly mutagenic if left unrepaired; it forms a stable base pair with adenine, resulting in G:C to T:A transversion mutations following DNA replication [77]. A critical enzyme of the base excision repair pathway, 8-oxoguanine DNA glycosylase 1 (OGG1), localized both in the mitochondria and nucleus of mature human spermatozoa, is able to excise 8OHdG, allowing the creation of an apurinic site and extracellular release of the adduct. However, human spermatozoa have a truncated base excision repair pathway as they lack the downstream components, apurinic endonuclease 1 (APE1), and X-ray repair complementing defective repair in Chinese hamster cells 1 (XRCC1) [78]. During oogenesis, the oocyte accumulates mRNA’s and proteins involved in base excision repair [79,80], and the apurinic sites in the sperm DNA can be repaired following fertilization by the zygote before the S-phase of the first mitotic division [81,82,83]. Although a remarkable acceleration of 8OHdG repair by the base excision repair pathway has been reported in the mouse oocyte following fertilization, OGG1 expression seems particularly low compared to male germ cells [77]. Therefore, spermatozoa carrying high levels of 8OHdG at the time of fertilization can undergo an incomplete DNA repair in the zygote, and this may impair the preimplantation embryo development [84] and fetal growth [85]. In addition, incomplete repair of sperm 8OHdG lesions has been linked to defects in offspring, including cancer and reduced lifespan [86,87]. Spermatozoa may also carry single- and double-strand DNA breaks (DSB), and the significance of the different methods to evaluate sperm DNA fragmentation has been recently reviewed elsewhere [88,89]. Oocytes should be able to repair such damage as they are equipped with components of DSB DNA repair, single-strand break (SSB) repair, and nucleotide-excision repair (NER) pathways [86]. However, female age has been shown to affect oocyte DNA repair pathways [90,91]. Further, detrimental effects of age have been reported on the DNA repair capacity of oocytes after IVF with X- irradiated sperm [92].

## 6. ROS Producers in Semen

The human ejaculate comprises mature and immature spermatozoa, germ cells, leukocytes, macrophages, and epithelial cells suspended in the seminal plasma. The main ROS producers in semen are leukocytes and immature spermatozoa. Peroxidase-containing leukocytes include macrophages (20–30%) and polymorphonuclear leukocytes (50–60%) and among them, neutrophils, which enter the semen in an activated state, are the most powerful ROS producers [93]. Seminal plasma antioxidants protect spermatozoa against the deleterious action of ROS generated by leukocytes [94], but in cases of infection or inflammation, the concentration of peroxidase-positive leukocytes can be ≥ 1 × 10^6^/mL, leading to leukocytospermia. In such conditions, peroxidase-positive leukocytes have been reported to produce 100-folds more ROS than under normal physiological conditions, and the antioxidant protection of seminal plasma becomes insufficient, leading to OS [95,96,97,98]. Although the association of leukocytospermia with male fertility in vitro is still under debate, the persistence of leukocyte contamination after removal of seminal plasma during sperm manipulation in ART has been reported to decrease the fertilization rates [98,99]. 

Leukocytes produce ROS much higher than spermatozoa, but their level in human semen is extremely low, and selection techniques such as density gradient centrifugation (DGC) are able to remove leukocytes from sperm suspensions. Spermatozoa and specific sperm subpopulations with enhanced ability to produce ROS are of concern once they are separated from seminal plasma and subjected to laboratory interventions. Although it would be important to determine which spermatozoon produces more ROS, these reactive molecules are extremely short-lived (10^−9^ s) and, most redox-sensitive probes measure OS rather than ROS directly [25]. 

A series of studies suggested a relationship between retention of the cytoplasmic droplet and human sperm membrane peroxidative damage using creatinine kinase (CK) and glucose-6-phosphate dehydrogenase (G6PDH) as markers of cytoplasm retention [100,101,102,103]. Gomez and collaborators [44] demonstrated an enhanced ROS production in human spermatozoa recovered from the low-density fraction after Percoll DGC characterized by large midpieces and high content of cytoplasmic enzymes. As G6PDH is required for the reduction of NADP^+^ to NADPH, it has been proposed to be involved in ROS production through membrane NADPH oxidase [104,105,106]. The enhanced ROS production by immature spermatozoa has been suggested to induce an OS in neighboring mature spermatozoa during their transit in the epididymis [44,107,108]. Koppers and collaborators reported that poorly motile and dysfunctional spermatozoa from the low-density region of Percoll gradients had an increased content of unsaturated fatty acids compared to motile and functional spermatozoa recovered from the high-density region, and this caused increased spontaneous production of mitochondrial ROS and consequent oxidative DNA damage in the mitochondria and nucleus [109]. Dysfunctional spermatozoa were characterized by the presence of excess residual cytoplasm, poor protamination, and retention of histones and have been suggested to result from defective differentiation during spermiogenesis [109]. Several studies showed a positive correlation between abnormal sperm morphology, ROS production, and DNA damage [93,107,110,111]. Moreover, an increased expression of the main isoform of NADPH oxidase (NOX), NOX5, an active membrane-bound generator of ROS in spermatozoa, has been reported in both teratozoospermic [112] and asthenozoospermic human ejaculates [44]. Positive correlations found between sperm morphological abnormalities, histone persistency, and lipid peroxidation indicate that abnormal spermatozoa with excessive histones and relaxed chromatin produce a higher amount of H_2_O_2_ [113]. The fact that the percentage of sperm persistent histones had an adverse effect on embryo development and clinical pregnancy outcomes is another indication of the incidence of sperm OS in such impairments [113]. 

The presence of an elevated fraction of dead spermatozoa is of concern during sperm manipulation and especially during cryopreservation in ART. Dead spermatozoa are generally considered high ROS producers [114] though their contribution to semen ROS generation can vary according to the species. The production of H_2_O_2_ by dead equine spermatozoa has been reported to be five-fold higher than in live spermatozoa [115]. Dead spermatozoa have been suggested to represent the major ROS producers through an aromatic L-amino acid oxidase (LAAO) pathway in bull semen [116]. In boars, the presence of dead spermatozoa in semen before and during freezing increased the production of ROS and sperm DNA fragmentation (SDF) in cryo-survived gametes [117]. L-amino acid oxidases have been reported in bull, ram, and stallion, and its activity is increased following sperm death [116,117,118,119,120]. Exposure of this enzyme to aromatic L-amino acids present in cryo-diluents and extenders upon loss of sperm membrane integrity promotes their deamination and the production of H_2_O_2_. Viable spermatozoa can therefore undergo a ROS-induced death promoting, in turn, the loss of viability in the remaining live cells [116,120]. Human spermatozoa containing LAAO are capable of inducing sperm capacitation and acrosomal exocytosis, but in contrast to bull and ram spermatozoa, LAAO activity is completely absent in nonviable human spermatozoa due to its rapid leakage after the loss of membrane integrity [121].

## 7. ART Procedures That Generate Sperm Oxidative Stress

**Figure 1:**
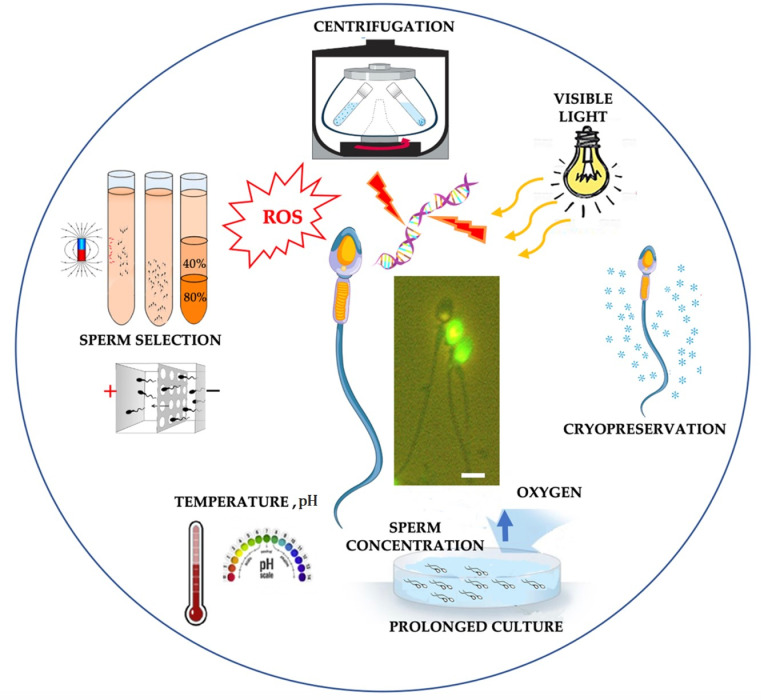
Factors inducing oxidative stress during sperm manipulation in ART. Central micrographs: TUNEL-labeled human sperm. Bar, 5 μm. Sperm drawing is modified from https://www.vecteezy.com/vector-art/1434164-human-sperm-or-spermatozoa-cell-structure (accessed date 3 March 2021).

Spermatozoa are subjected to a wide range of in vitro manipulations during ART in both human and domestic animals. In a typical ART setting, the potential sources of oxidative stress in vitro include endogenous and exogenous factors. Although gametes themselves have the potential to generate ROS, OS during ART could also arise from several exogenous factors such as exposure to visible light, centrifugation, cryopreservation, culture media, O_2_ tension, pH, and temperature (Figure 1).

In vitro manipulation of sperm primarily involves the separation of cells from the seminal plasma that contains several protective enzymatic and non-enzymatic antioxidants and low molecular weight compounds exerting powerful antioxidant activity [122,123]. On the other hand, as the main sources of intracellular ROS in semen are leukocytes [124,125,126] and immature sperm with abnormal head morphology and cytoplasmic retention [44,70], removal of these ROS sources through sperm selection can reduce SDF and other oxidative damage.

Whole semen can be subjected to several sperm selection procedures. The most commonly applied are swim-up techniques and centrifugation through discontinuous density gradients of silane-coated silica colloidal particles. Swim-up can be performed from the whole semen or a pellet obtained through centrifugation, through stratification of a medium containing bicarbonate and albumin, in which motile sperm migrate.

Several factors could generate OS during the swim-up from a pellet. In fact, (1) the seminal plasma antioxidants are removed as semen is mixed with culture medium; (2) the shearing forces induced on spermatozoa and cells during centrifugation result in the production of ROS [125]; (3) before migration, spermatozoa remain tightly packed for a variable time in the pellet along with leukocytes and abnormal spermatozoa which are both ROS producers. The level of ROS generation is dependent on the severity and duration of exposure to centrifugation force [127,128]. Preparation techniques involving the centrifugation of unfractionated sperm suspensions, such as simple washings or swim-up from the pellet, have been associated with a sudden burst of ROS production, reduced motility, and impaired sperm-oocyte fusion in the zona-free hamster oocyte penetration test [125,129]. Therefore, it has been suggested to adopt sperm-selection techniques in which centrifugation is only applied after the sperm motile fraction has been selected, as in the swim-up from semen and gradient centrifugation procedures. 

Several studies assessed the effects of DGC on DNA oxidative damage analyzing SDF through TUNEL and the formation of the DNA-base adduct 8-hydroxy-2′-deoxyguanosine. Aitken et al. showed that a majority of TUNEL and 8OHdG positive cells were dead both before and after Percoll and Puresperm, and DGC positively selected motile and viable cells, reducing the proportion of TUNEL-positive cells. However, DGC increased the fraction of viable cells with high levels of 8OHdG both in a patient and donor population, leading to an increase of viable TUNEL-positive sperm only in the former [25]. Such effects were attributed to ROS production during centrifugation through the dense gradient rather than to the simple act of centrifugation that can also increase ROS generation [125,129]. Iwasaki and Gagnon [130] showed a four- to five-fold increase of ROS in Percoll-washed spermatozoa compared to the original semen sample, whereas a reduction of ROS was reported in spermatozoa recovered after DGC in SpermGrad™ [131], and a lack of increase in sperm DNA damage was reported after centrifugation in PureCeption™ and Isolate™ [132]. Zhao et al. reported a significant reduction of ROS and SDF after the selection of normozoospermic samples through PureCeptionTM density gradient centrifugation or swim-up [133]. 

The induction of oxidative DNA damage in PureSperm recovered spermatozoa was demonstrated to depend on the simple exposure to the density medium and not associated with generalized oxidative stress. Contaminating metals found in Percoll, PureSperm, and other commercial density media have been suggested to promote ROS generation in the immediate vicinity of DNA, and the addition of EDTA to PureSperm fully reversed its ability to induce oxidative DNA damage [134]. Muratori and collaborators reported that PureSperm centrifugation increased the percentage of DNA fragmented spermatozoa in about 50% of subjects studied, while a reduction was observed in the remaining ones. Interestingly, the patients with PureSperm-induced DNA oxidation had a 50% lower chance of achieving pregnancy after IVF/ICSI [135], even though no differences were found after basal semen analysis. In a subsequent paper, the effects of DGC and swim-up selection on the TUNEL positive live and dead fractions were evaluated in male partners of infertile couples, which did not show any differences in average pre- and post-selection SDF values but again a different behavior according to the sample. The analysis of single samples revealed that in some subjects, both selection methods induced SDF, whereas in other subjects, the opposite finding was observed. The analysis of viable-DNA fragmented sperm allowed the identification of additional subjects undergoing DNA damage during selection with respect to conventional methods revealing total SDF. Moreover, under these conditions, the increase of both total and viable SDF (*p* = 0.047) in samples processed by DGC was higher than swim-up of good quality semen samples and about equal to swim-up selection in case of poor-quality samples [136]. Although ROS were not assessed in these studies, the occurrence of DNA fragmentation in spermatozoa after ejaculation is generally considered a result of OS.

Apparently, controversial findings of studies aimed to evaluate the effects of discontinuous gradient centrifugation on ROS production and/or DNA damage could be explained by the particular gradient used, presence of metal contamination, and chelating agents in diluting media, as well as by semen quality and patient age.

More advanced sperm selection techniques, including magnetic cell sorting (MACS) with annexin V conjugated beads, intracytoplasmic morphologically selected sperm injection (IMSI), and physiological ICSI using hyaluronic acid binding (PICSI), have been proposed to select high-quality spermatozoa with intact chromatin [137,138,139,140]. However, the additional value of such selection procedures on ART outcome is still debated [141,142], and importantly, these techniques require longer culture or handling time, pH, and temperature variations, and exposure to visible light under the microscope which could induce OS and consequent DNA damage. 

Ashgar et al. developed a centrifugation-free and flow-free microfluidic platform where spermatozoa from whole semen migrate against gravity through 3, 5, or 8 μm pores carbonate filters [143]. Human semen processed through the platform had decreased ROS generation and SDF compared to parallel spermatozoa recovered through swim-up from pellet [143]. Ebner and collaborators found a 90% reduction of SDF after a centrifugation-free selection in special chambers, Zech-selectors, whereas no reduction was achieved after DGC [144]. Other studies evaluated the efficiency of methods based on sperm electric charge in selecting DNA fragmentation-free spermatozoa. A significant reduction of SDF through a Zeta potential selection on spermatozoa recovered by DGC compared to DGC alone has been recently reported [145]. An electrophoresis method developed to select high-quality human spermatozoa on the basis of their high negative charge [146] was shown to efficiently select spermatozoa without oxidative DNA damage in contrast to Percoll DGC that increase such damage compared to neat semen [147]. In addition, Simon and collaborators showed that DGC increased the fraction of positively charged and decreased that of negatively charged human spermatozoa compared to neat semen [148]. 

Several studies showed that prolonged sperm incubation in culture media leads to OS, SDF, and DNA oxidation in human and animal models [38,148,149,150,151]. Incubation of spermatozoa from healthy donors after selection through swim-up from pellet led to a reduction in motility and increased ROS levels starting from 6 h and reaching a peak at 48 and 24 h, respectively [152]. Other studies found significant changes at earlier incubation times. In a study on 24 normozoospermic and 20 oligozoospermic patients, washed spermatozoa in 75% of the patients decreased total and progressive motility and increased lipid peroxidation and SDF during incubation for 6 h. [153]. Nabi and collaborators found an increased SDF in spermatozoa selected through swim-up from whole semen during incubation for 3 h (from 4.38% at 0 h to about 11% at 3 h) [154]. Sperm concentration during in vitro manipulation also affects viability, ROS levels, and SDF in different species. Post-thaw incubation of ram spermatozoa at 6 to 100 × 10^6^/mL for 6 h resulted in an increase of SDF, with lower sperm concentrations being safer in this respect. The increase of SDF also depended on individual ram semen [155]. The sperm concentration during storage of bull semen in a fresh extender also affects the ROS levels, with higher concentrations of spermatozoa (5 × 10^6^ per 0.25 mL artificial insemination dose) exerting detrimental effects on sperm cell viability and increased OS compared to lower sperm concentrations [156]. In our lab, incubation of washed human spermatozoa from seven normozoospermic patients at serial dilutions ranging from 5 to 100 × 10^6^/mL in Sydney IVF Gamete Buffer for 3 h at 37 °C led to increased SDF and oxidative DNA damage except for concentrations of 5–10 × 10^6^/mL (Gualtieri and Talevi, unpublished data).

The effects of prolonged incubation on ROS production could also depend on the specific sperm handling medium used. In fact, incubation of DGC selected human spermatozoa for 3 h in four commercial sperm-washing buffers resulted in different levels of ROS production, sperm viability, and capacitation-associated tyrosine phosphorylation and membrane reorganization. Impairment of sperm function can occur in media with high ROS in response to OS, and in media with very low levels of ROS in which reductive stress can prevent the ROS-induced capacitation events in spermatozoa [157]. Measurements of oxidation-reduction potential (ORP) of sperm processing media using male infertility oxidative system (MiOXSYS) showed values ranging from a condition of reductive stress in freezing media designed to counteract the OS induced by cryopreservation to a condition of OS in sperm washing media. Such measurements could help to define the correct values of sperm processing media to ensure the development of ROS-induced physiological events without introducing an OS during sperm preparation [158]. 

Oxygen tension during sperm media incubation could also influence the levels of ROS and their beneficial or detrimental effects on spermatozoa. Although spermatozoa are generally incubated in 20% O_2_, samples with high ROS levels could benefit from reduced O_2_ tensions. In fact, no differences in sperm function were detected for normozoospermic sperm samples after incubation under 5 versus 20% O_2_, whereas 5% O_2_ incubation was found beneficial for oligozoospermic samples that are known to have higher levels of ROS than fertile samples [159]. 

Moreover, incubation of spermatozoa in whole semen or after washing/selection at 37 °C accelerates the DNA damage compared to room temperature. In fact, incubation of fresh liquified semen for 1 h at 37 °C has been reported to significantly increase phosphatidyl serine translocation and TUNEL positivity compared to parallel samples maintained at 34 and 25 °C [160], whereas incubation of DGC and swim-up selected spermatozoa at 37 °C increases the oxidative damage that can be minimized storing samples at room temperature [161,162]. 

Exposure of cells to light, especially to short wavelengths in the UV range but also to violet-blue wavelengths, is considered potentially harmful in terms of ROS production and DNA damage. Phototoxicity depends on the wavelength, photon concentration, and also on the cell type, and transmitted light microscopy is generally regarded as poorly phototoxic to cells [163,164,165]. Although the exposure of gametes and embryos to light during microscope observation is generally reported as a potential exogenous source of ROS production [166,167], specific studies on the detrimental effects caused by exposure of spermatozoa to light are lacking. On the other hand, photo-modulation with visible light at different wavelengths has been used to increase sperm hyperactivation and fertilizing potential of human and animal spermatozoa, leading to intracellular Ca^2+^ rise and a controlled mitochondrial ROS production without affecting the DNA [168,169,170]. 

Sperm cryopreservation is routinely used in human ART and in the preservation and transport of male gametes in most domestic species. However, several studies in human and domestic animals indicate that cryopreservation reduces sperm viability and can impair sperm motility, mitochondrial activity, chromatin integrity, and reproductive potential in surviving cells [114,171,172,173,174].

Although the high number and quality of gametes present in good-quality ejaculates make survival rates acceptable, spermatozoa in poor-quality ejaculates can be more susceptible to cryoinjuries impairing the recovery of viable spermatozoa after thawing in severely oligozoospermic patients [175]. In fact, impairment of sperm motility, DNA integrity, and sperm competence, as a result of cryopreservation, has been reported, especially in subfertile and infertile men [176,177,178]. There is a general agreement on the contribution of OS as the main factor accounting for the reduced survival and competence of cryopreserved spermatozoa in supporting fertilization and embryo development, especially in non-human mammals [85,150,179,180]. Hence, several studies have been addressed to understand the causes and remedies of cryopreservation-associated sperm damage and the effects of pre- or post-treatment with antioxidants on the improvement of cryopreserved spermatozoa in infertile patients and in animal species prone to cryodamage. The most commonly assessed damages considered are DNA fragmentation and oxidation, ROS levels, and collapse of inner mitochondrial membrane potential [181]. Mitochondria are thought to be central in triggering the OS associated with sperm cryopreservation and opening of the mitochondrial permeability transition pore in response to the increase of intracellular Ca^2+^ brought about by permeant cryoprotectants has been proposed to represent the main mechanism in this respect (see [182] and references therein for review).

## 8. Effects of Sperm OS on Embryo Development

Studies have ascertained that OS in spermatozoa can substantially contribute to the poor embryo development in experimental animal models [180,183,184] as well as in humans undergoing ART [185] (Table 1). The most predominant consequence of OS on spermatozoa is DNA damage. Therefore, most of the studies in the literature have correlated the embryonic response to the sperm OS with reference to the sperm DNA damage. The response to the entry of spermatozoa carrying DNA damage to the ooplasm during fertilization can have various consequences [81,82,186,187,188,189]. Depending on the extent of sperm DNA damage, the oocyte can repair the damage in spermatozoa and lead to a healthy embryo; it can successfully fertilize with the development of the defective embryo, or it may lead to fertilization failure or embryo arrest [190]. Simões et al. have demonstrated that elevated endogenous oxidative stress and DNA damage in bovine spermatozoa give rise to blastocysts with a high percentage of apoptotic cells [191]. Similar observations were made in studies in which OS was exogenously induced. Bittner et al. observed that the spermatozoa exposed to H_2_O_2_ resulted in embryos carrying high DNA damage at the early cleavage stage as well as blastocyst stage [184]. De Castro et al. reported a dose-dependent decrease in cleavage and blastocyst rates when H_2_O_2_ exposed bull spermatozoa were used for in vitro fertilization [183]. In vitro exogenous OS, induced through xanthine/xanthine oxidase system, has been shown to decrease frozen–thawed bull sperm motility, increase SDF, reduce fertilization rates, and reduce blastocyst rates and quality. Pre-treatment with zinc, d-aspartate, and co-enzyme Q10 before exogenous OS was able to prevent these effects [179]. Lane et al. have reported that inducing oxidative stress in mouse spermatozoa using H_2_O_2_ can cause oxidative damage to sperm, which not only reduced the developmental potential of preimplantation stage embryos but also decreased their implantation potential. The OS in spermatozoa appeared to have a sex-specific effect on the growth of offspring and their metabolic function. Further, female offspring had metabolic disturbances such as glucose intolerance and increased levels of adipose tissues [85]. Wyck et al. have demonstrated that the sperm OS can impair the epigenetic reprograming in early cattle embryos due to impaired active DNA demethylation on male pronucleus, which can potentially contribute to defective embryo development and poor embryo quality [19]. Rhesus embryos produced from sperm with ROS exposure prior to fertilization exhibit early cleavage abnormalities and a delayed start of first cytokinesis compared to control embryos [192]. Few clinical studies have tried to establish the relationship between basal OS in spermatozoa/seminal plasma and the reproductive outcome in clinical settings. Elevated ROS in seminal plasma has a negative correlation with fertilization rate in IVF and ICSI [193]. Similarly, excessive ROS in semen was found to reduce the fertilization rate, embryo quality, and live birth rate [194]. Further, Ghaleno et al. have shown that high levels of both sperm intracellular H_2_O_2_ and O_2_^•−^ in ICSI patients have a deleterious effect on sperm mitochondrial membrane potential; however, only H_2_O_2_ may interfere in pronuclear formation [185]. Further, oxidative DNA damage in human spermatozoa can influence time to pregnancy since an inverse relationship between sperm DNA oxidation and monthly fecundity rate was observed in a naturally conceiving population [195]. In contrast to these observations, Pujol et al. did not find any significant relationship between embryo quality and OS levels in the ejaculate. Hence, these authors have not recommended the routine OS measurement in fresh ejaculate for all patients, especially when the oocytes for the ART cycle come from women younger than 35 years of age [196]. 

Oxidative stress-induced protein damage in sperm is expected to be implicated in embryo degeneration at a progressive phase of pregnancy, which could lead to pregnancy loss. Levels of SDF, ROS, and total antioxidant capacity (TAC) have significant correlations with recurrent spontaneous miscarriage. The increase in SDF, along with the increase of free radicals and reduction of total antioxidants in semen, has increased the frequency of repeated miscarriages [197]. It has been suggested that histone-carrying sites for oxidative modification such as arginine and lysine might be responsible for disturbing the paternal epigenomic control during early stages of embryonic differentiation, leading to pregnancy loss [198]. 

Fewer studies were focused on the effects of OS induced by specific sperm manipulations during ART on embryo development. Simon and collaborators showed that selection of human spermatozoa through DGC increased the fraction of positively charged and decreased that of negatively charged spermatozoa compared to neat semen. The negatively charged fraction was relatively free of DNA damage and positively associated with increased fertilization and blastocyst rates. In addition, embryos generated by patients with a higher fraction of negatively charged spermatozoa had better implantation and clinical pregnancy rates [148].

A recent study assessed the effects of cushioned centrifugation of frozen–thawed bull spermatozoa using a commercial colloidal iodixanol-based solution (Cushion Fluid, Minitube^®^, Tiefenbach, Germany) on fertilization and embryo development. The addition of cushion fluid at the bottom of a Percoll gradient before centrifugation (C1), as well as during washing of recovered spermatozoa (C1–2), or exclusively at washing (C2), was compared with Percoll centrifugation without a cushion fluid (C). ROS levels in C and C1 were comparable and were significantly lower than in C1–2 and C2 treatments. Interestingly, cushioned Percoll centrifugation (C1) produced higher fertilization, cleavage, and blastocyst rates compared to conventional Percoll centrifugation (C) [199].

Conflicting results have been reported on the effects of sperm selection through hyaluronic acid compared to polyvinylpyrrolidone (PVP) prior to ICSI on ART outcome [138,200,201,202]. Although measurements of oxidation-reduction potential (ORP) of human spermatozoa treated with PVP, hyaluronic acid, or medium indicate that PVP provides higher antioxidative protection, exposure of human spermatozoa to the different handling media do not affect mouse oocyte activation, and injection of the three media in parthenogenetically activated mouse oocytes had no effects on embryo development [203].

Spontaneous OS during culture was investigated in human spermatozoa and in bovine frozen-thawed spermatozoa. In both species, culture for 6 and 3 h, respectively, decreased total and progressive motility and increased lipid peroxidation and SDF, and such effects were prevented by supplementation of sperm media with zinc, d-aspartate, and co-enzyme Q_10_ [150,153]. Moreover, in the bovine, blastocyst rate was found to be significantly higher in oocytes fertilized by treated spermatozoa, and these blastocysts harbored a significantly lower percentage of apoptotic cells compared to parallel spermatozoa incubated in the medium alone [150]. 

More studies are available in the literature on the detrimental effects of sperm cryopreservation on embryo development after IVF or ICSI. An interesting study considered the effects of cryopreserved versus fresh human spermatozoa on embryo development after ICSI in normal or dysmorphic oocytes. A negative influence of sperm cryopreservation on the quality of cleavage stage embryos and on blastocyst rates was only detected when one oocyte defect was present, suggesting that healthy oocytes can better repair damaged paternal DNA [204]. Mouse sperm cryopreservation increases SDF and affects embryo development according to the specific cryopreservation procedures adopted [205]. In bovine, treatment of frozen–thawed spermatozoa with GSH before ICSI improved the rates of embryos reaching the 4–8-cell stage and blastocyst stage [206].

Sperm sorting, a valuable technique for selecting desired sex in domestic animals, is associated with increased OS and SDF [207]. Bovine embryos derived from sex-sorted spermatozoa had an increased incidence of arrest at the 4-cell stage, and reduced survival and blastocyst rates [208]. Supplementation of vitamin C or lycopene in washing and fertilization medium improves the fertilization, cleavage, and blastocyst rates of oocytes inseminated with sex-sorted bull sperm [209]. 

**Table 1:** Effects of endogenous, experimentally induced, and art-associated oxidative stress on sperm function, embryo development, and reproductive outcome.

Oxidative Stress	ART Treatment	Species	Subjects or Samples	Experimental Groups	Adverse Effects: Sperm Function	Adverse Effects: Embryo Development	Antioxidant (Effect)	Refs
Endogenous	NA	Human	First pregnancy—	High vs. low 8OHdG	NA	Natural conception rate ↓	NA	[195]
			planning males					
Endogenous	ICSI	Human	Fresh semen	High vs. low ROS	Viability ↓	Fertilization rate ↓	NA	[194]
					Motility ↓	Pregnancy rate ↓		
					Morphology ↓			
					DNA integrity ↓ (TUNEL)			
Endogenous	IVF/ICSI	Human	Fresh semen	High vs. low ROS	Vitality ↓	Fertilization rate ↓	NA	[193]
					Membrane integrity ↓			
					Morphology ↓			
Endogenous	Washing/IVF	Bovine	F/T semen	High vs. low TBARS	DNA integrity↓ (SCSA)	Cleavage ↓		
				bull semen		Blastocyst DNA fragm. ↑	NA	[191]
						(TUNEL)		
Endogenous	Swim-up/ICSI	Human	Fresh semen	High vs. low ROS	ΔΨm ↓		NA	[185]
				High H_2_O_2_ vs. high O_2_^•−^	ΔΨm ↓	2 pronuclei rate ↓		
Endogenous	Swim-up/ICSI	Human	F/T semen	High vs. low O_2_^•−^	NA	None	NA	[196]
				ICSI donor oocytes				
Endogenous	NA	Human	Fresh semen	Idiopathic recurrent	Motility ↓	Recurrent pregnancy loss	NA	[198]
				pregnancy loss male	Lipid peroxidation ↑ (TBARS)			
				partners vs. fertile men	Protein carbonylation ↑			
					Histone retention (ABS) ↑			
Endogenous	NA	Human	Fresh semen	Idiopathic recurrent	Motility ↓	Recurrent pregnancy loss	NA	[197]
				pregnancy loss male	ROS ↑ (luminol)			
				partners vs. fertile men	TAC ↓			
					DNA integrity↓ (SCSA, TUNEL)			
Ind. (H_2_O_2_)	Culture 1h/IVF	Mouse	Fresh semen	H_2_O_2_ vs. medium	ROS ↑ (carboxy-DCFDA)	8-cell rate ↓	NA	[85]
					Mitochondrial ROS ↑(MSR)	Blastocyst rate ↓		
					Lipid peroxidation ↑(4-HNE)	Implantation rate ↓		
						Fetal weight ↓		
						Fetal:placental ratio ↓		
						Crown-rump length ↓		
						Female offspring health ↓		
Ind. (H_2_O_2_)	Culture 1h/IVF	Bovine	F/T semen	H_2_O_2_ vs. medium	Motility ↓	Cleavage ↓	NA	[19]
					DNA integrity ↓(SCSA)	Blastocyst rate ↓		
						Active DNA demethylation		
						paternal pronucleus ↓		
Ind. (X-XO)	Culture 2h/ICSI	Rhesus	F/T semen	X-XO vs. medium	Motility ↓	Cleavage ↓	NA	[192]
						Delayed first cytokinesis ↑		
						Blastocyst rate ↓		
Ind. (H_2_O_2_)	Culture 1h/IVF	Bovine	F/T semen	H_2_O_2_ vs. medium	Motility ↓	Cleavage ↓	NA	[183]
					ROS↑ (CellROX™)	Blastocyst rate ↓		
					DNA integrity↓ (AO)			
Ind. (X-XO)	Culture 3h/IVF	Bovine	F/T semen	X-XO vs. medium vs	Motility ↓	Cleavage ↓	Zn, D-asp, CoQ_10_	[179]
				antioxidants + X-XO	DNA integrity↓ (TUNEL)	8-cell rate ↓	(protection)	
						Blastocyst rate ↓		
						Blastocyst DNA fragm. ↑		
						(TUNEL)		
Ind. (H_2_O_2_)	Culture 1h/IVF	Bovine	F/T semen	H_2_O_2_ vs. medium	Motility ↓	Delayed first cleavage ↑	NA	[184]
					DNA integrity↓ (SCSA)	Cleavage ↓		
						Blastocyst rate ↓		
						Blastocyst DNA fragm. ↑		
						(Comet, TUNEL)		
Ext. culture	Culture 3h/IVF	Bovine	F/T semen	Antioxidants	Motility 1 h ↑	Cleavage ↑	Zn, D-asp, CoQ_10_	[150]
				vs medium	DNA integrity 3 h ↑ (TUNEL)	8-cell rate ↑	(protection)	
						Blastocyst rate ↑		
						Blastocyst DNA fragm. ↓		
						(TUNEL)		
DGC + mEP	IVF/ICSI	Human	Fresh semen	DGC vs. fresh semen	Neg. charged sperm (NCS) ↓	NCS ↑: IVF fertilization rate ↑	NA	[148]
					Pos. charged sperm (PCS) ↑	NCS ↑: blastocyst rate ↑		
					NCS: DNA integrity (TUNEL) ↑	NCS ↑: implantation rate ↑		
					PCS: DNA integrity (TUNEL) ↓	NCS ↑: clin. preg. rate ↑		
					NCS ↑: Histone retention (ABS) ↑			
DGC	IVF	Bovine	F/T semen	DGC + cushioning	ROS (DCHF-DA): no differences	Fertilization rate ↓	NA	[199]
				vs DGC	Motility: no differences	Cleavage ↓		
Immobiliz.	Mouse oocyte	Human	Fresh semen	PVP vs. HA vs	ORP: PVP < HA < medium	MOAT: no differences	NA	[203]
	activation			medium				
	(MOAT)							
Cryopr.	IVF	Mouse	Fresh and	F/T vs. fresh semen	DNA integrity ↓ (SCSA)	2-cell embryos ↓	NA	[205]
			F/T semen			Blastocyst rate ↓		
Cryopr.	ICSI normal	Human	Fresh and	F/T vs. fresh semen	NA	ICSI defective oocytes:	NA	[204]
	and defective		F/T semen			Day 2, 3 embryo quality ↓		
	oocytes					Blastocyst rate ↓		
Cryopr.	ICSI	Bovine	F/T semen	No GSH vs. post-thaw	Motility ↓	Cleavage rate ↓	GSH	[206]
				GSH treatment	ΔΨm ↓	Blastocyst rate ↓	(protection)	
					ATP ↓			
Sex sorting	IVF	Bovine	F/T semen	Post- vs. pre-sorting	Motility ↓	Cleavage rate ↓		[208]
					Hyperactivation ↓	4-cell rate ↓		
					Survival ↓	Blastocyst rate ↓		
					(Extent is bull specific)	Embryo survival ↓ (extent is		
						bull specific)		
Sex sorting	IVF	Bovine	F/T semen	Washing/fertilization	MDA↓	Cleavage rate ↑	Vitamin C or	[209]
				with vs. without	Viability ↑	Lyc: Blastocyst rate ↑	Lycopene	
				Vitamin C (VC) or Lycopene (Lyc)	Apoptosis (Annexin V) ↓		(protection)	
					ΔΨm ↑ (VC: extent bull specific)			

Effects of endogenous, experimentally induced (Ind.), and ART-associated oxidative stress on sperm function, embryo development, and reproductive outcome. ABS, aniline blue staining; AO, acridine orange staining; carboxy-DCFDA, 5- and 6-carboxy-2’,7’-dichlorofluorescein diacetate; CoQ10, coenzyme Q10; Cryopr., cryopreservation; D-asp, D-aspartate; DCHF-DA, 2ʹ,7ʹ-dichlorofluorescin diacetate; DGC, density gradient centrifugation; ΔΨm, inner mitochondrial membrane potential; Ext., extended; Fragm., fragmentation; GSH, reduced L-glutathione; HA, hyaluronic acid; 4-HNE, 4-hydroxynonenal; Immobiliz., immobilization; MDA, malondialdehyde; mEP, micro-electrophoresis; MSR, MitoSOX™ Red; NA, not applicable; 8OHdG, 8-hydroxy-2′-deoxyguanosine; ORP, oxidation-reduction potential; ROS, reactive oxygen species; SCSA, sperm chromatin structure assay; TBARS, thiobarbituric acid reactive substances assay; TAC, total antioxidant capacity; Zn, zinc; ↑, increase; ↓, decrease.

## 9. Conclusions

Several ART manipulations have the potential to induce an ex novo OS in spermatozoa. The individual semen characteristics can influence the degree of OS during sperm in vitro manipulations. Although routinely used sperm selection methods can enhance the recovery of spermatozoa with higher DNA integrity compared to the neat semen, an enhancement of DNA fragmentation or oxidation can be found in some individuals. The induction of OS and the consequent enhancement of DNA damage could be minimized using advanced selection procedures such as microelectrophoresis, Zeta potential, and microfluidic methods. However, such technologies still remain rarely applied in clinics.

Despite substantial evidence in human and animal models that clearly indicate that sperm OS exerts detrimental effects on embryo development, fetal growth, and postnatal health, studies addressing the effects of specific in vitro manipulations on sperm OS and its consequences on embryo development and ART outcome are limited. Treatment with specific antioxidants in vitro could prevent the impairment of sperm function and competence induced by ART manipulations. However, their application in the clinical practice is still limited and should be introduced, taking into account the extent of OS caused by the specific ART procedures and the need for a personalized treatment according to the semen characteristics of the patient. Indeed, the prevention of OS could be needed in some patients in whom ROS production exceeds semen antioxidants’ defenses, whereas it could be dangerous in patients with low ROS levels. This could lead to a reductive stress condition that suppresses the physiological redox signaling needed for sperm capacitation, hyperactivation, acrosome reaction, and sperm–oocyte fusion.

During the next few years, the increase of success rate and safety of ART will require further research to develop and individualize more adequate techniques to avoid the iatrogenic causes of sperm OS and its short- and long-term consequences on the reproductive outcome.
